# A Beckwith–Wiedemann-Associated *CDKN1C* Mutation Allows the Identification of a Novel Nuclear Localization Signal in Human p57^Kip2^

**DOI:** 10.3390/ijms22147428

**Published:** 2021-07-11

**Authors:** Emanuela Stampone, Debora Bencivenga, Clementina Barone, Marilena Di Finizio, Fulvio Della Ragione, Adriana Borriello

**Affiliations:** Department of Precision Medicine, University of Campania “Luigi Vanvitelli”, 80138 Naples, Italy; emanuela.stampone@unicampania.it (E.S.); debora.bencivenga@unicampania.it (D.B.); clementina.barone@unicampania.it (C.B.); marilena.difinizio@unicampania.it (M.D.F.)

**Keywords:** Beckwith–Wiedemann syndrome, *CDKN1C* mutations, NLS, p57^Kip2^, R316W-p57^Kip2^

## Abstract

p57^Kip2^ protein is a member of the CIP/Kip family, mainly localized in the nucleus where it exerts its Cyclin/CDKs inhibitory function. In addition, the protein plays key roles in embryogenesis, differentiation, and carcinogenesis depending on its cellular localization and interactors. Mutations of *CDKN1C*, the gene encoding human p57^Kip2^, result in the development of different genetic diseases, including Beckwith–Wiedemann, IMAGe and Silver–Russell syndromes. We investigated a specific Beckwith–Wiedemann associated *CDKN1C* change (c.946 C>T) that results in the substitution of the C-terminal amino acid (arginine 316) with a tryptophan (R316W-p57^Kip2^). We found a clear redistribution of R316W-p57^Kip2^, in that while the wild-type p57^Kip2^ mostly occurs in the nucleus, the mutant form is also distributed in the cytoplasm. Transfection of two expression constructs encoding the p57^Kip2^ N- and C-terminal domain, respectively, allows the mapping of the nuclear localization signal(s) (NLSs) between residues 220–316. Moreover, by removing the basic RKRLR sequence at the protein C-terminus (from 312 to 316 residue), p57^Kip2^ was confined in the cytosol, implying that this sequence is absolutely required for nuclear entry. In conclusion, we identified an unreported p57^Kip2^ NLS and suggest that its absence or mutation might be of relevance in *CDKN1C*-associated human diseases determining significant changes of p57^Kip2^ localization/regulatory roles.

## 1. Introduction

Human p57^Kip2^ (hereinafter p57) is a 316 amino acid protein belonging to the CIP/Kip (CDK Interacting Protein/Kinase inhibitory protein) family. The protein was identified by sequence homology with the other two members of the family, i.e., p21^Cip1/WAF1^ and p27^Kip1^ [1,2]. Differently from p21^Cip1/WAF1^ and p27^Kip1^, scarce information has been accumulated on p57 structural characteristics despite more than 25 years having passed since its initial identification [3,4,5,6,7,8]. Certainly, what has definitely been demonstrated is that all CIP/Kip members present some structural features that are necessary to exert their function in the regulation of the cell division cycle. Particularly, based on primary sequence similarities, these proteins share the so-called KID domain (Kinase Inhibitor Domain) at the amino terminal region. The KID is required to bind the Cyclin-CDKs complexes and to induce the kinase inhibition [1,2,9]. Besides this inhibitory activity, p57 and its siblings have been reported as being able to promote the assembly of the Cyclin D-CDK4/6 complex, a striking characteristic that allows them to be considered more comprehensively as major cell cycle regulators [6,7,8,9]. The N-terminal domain is also involved in the interaction with transcription factors such as b-Myb and MyoD, controlling gene expression [5]. In contrast, they exhibit strongly divergent central and carboxy-terminal domains. Interestingly, this divergence might also involve mammal orthologs of the same CIP/Kip protein (for example, human p57 vs. mouse p57). The central part of human p57, consisting of proline/alanine-rich repeats (PAPA region), is absent in the other CIP/Kip members and probably also justifies the difference between the apparent molecular weight of the protein (i.e., 57 kDa) observed on SDS-polyacrylamide gel electrophoresis and that determined on the basis of the residue composition (namely, about 32 kDa). In this unique central p57 region, a site of interaction with LIMK1, a kinase involved in the modulation of actin polymerization, has been reported [10,11]. In the p57 C-terminal region, a QT domain is recognizable that is rich in glutamine and threonine residues, similar to that of p27^Kip1^ and putatively important in protein–protein interaction [12], and a PCNA (Proliferating Cell Nuclear Antigen) binding region, homologous to that of p21^Cip1/WAF1^, required for preventing DNA replication [13,14]. Moreover, based on the sequence homology of human p57 with its mouse counterpart and with human p27^Kip1^, it has been hypothesized that the C-terminal region contains a putative nuclear localization signal (NLS) that is necessary for its nuclear function [1]. Particularly, it has been reported that the truncation of mouse p57 through the removal of the C-terminal domain determines the exclusion from the nucleus, indirectly confirming the hypothesis that active nuclear import is mediated by a putative NLS similar to that demonstrated for p27^Kip1^ [15,16].

It has been established that p57 is crucial during embryogenesis due to the observation that *Cdkn1c* (the gene encoding mouse p57) ablated mice mostly die after birth and that those surviving develop numerous organ abnormalities [17,18]. In addition, it has been found that mutations in the *CDKN1C* sequence in humans are responsible for the pathogenesis of growth disorders, such as Beckwith–Wiedemann (BWS), IMAGe (Intrauterine growth restriction, Metaphyseal dysplasia, Adrenal hypoplasia congenita, and Genital anomalies), and Russell–Silver (RSS) syndromes [19]. Particularly, a percentage of patients with BWS carry missense or frameshift mutations of *CDKN1C* [20,21]. More in detail, it has been reported that the majority of the missense mutations determine the loss of function of the protein by affecting the KID domain sequence and the N-terminal region [4]. In addition, numerous p57 frameshift mutations were identified in BWS patients along the entire *CDKN1C* sequence [4]. Those mutants, even those retaining the ability to bind the Cyclin/CDKs complexes, might lack the C-terminal domain containing the specific site of these interactions and/or the putative NLS. In the latter case, they might actually be inactive since they are unable to enter the nucleus and perform their CDK-dependent and independent functions [6,22]. Thus, the precise experimental identification of p57 NLS is of great relevance in mechanistic studies on BWS *CDKN1C* mutants. In this context, when we analysed the missense mutations identified in BWS patients, we were surprised to find a change (c.946 C>T) involving the last triplet of the coding sequence of *CDKN1C* [23]. In particular, this genetic change consists of the substitution of the arginine in position 316 with a tryptophan (R316W-p57) [23]. As a matter of fact, the residue position should not affect KID activity, protein interaction, or localization. However, since the mutation occurs in a basic domain typical of an NLS [24], we decided to investigate the effect of the genetic change on variant protein localization. The results of the study allowed for the identification of an undescribed p57 sequence required for nuclear localization.

## 2. Results

### 2.1. Effects of R316W-p57 Transfection on Cell Proliferation Rate and Cell Cycle Phase Distribution

**Figure 1:**
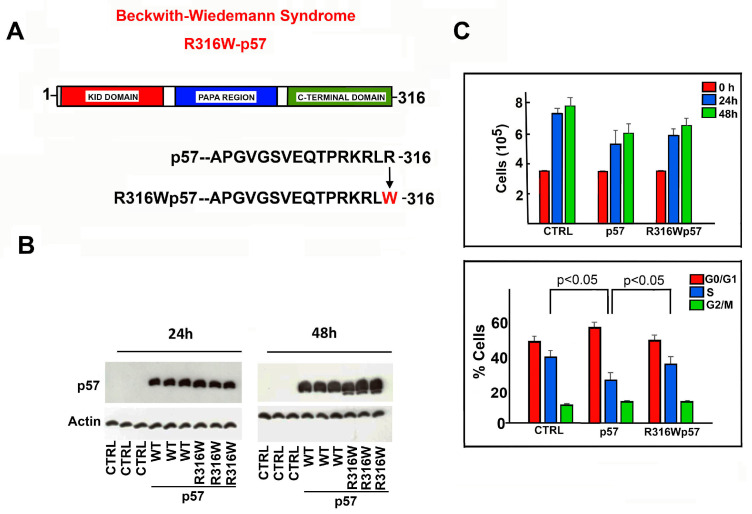
R316W-p57 mutation: effect on cell proliferation and cell cycle phase distribution. (**A**) Schematic representation of the structural domains of human p57 reporting the BWS-associated R316W mutation under analysis. (**B**) WB analysis of p57 in protein extracts of HEK293 cells transfected in triplicates with the empty vector (indicated as CTRL) and the plasmids encoding human WT- and R316W-p57 for 24 h and 48 h. Actin content was used to verify loading of equal amounts of proteins. (**C**) The graphs show the effect of the exogenous WT- and R316W-p57 with respect to CTRL on cell proliferation rate (upper panel) and on cell cycle phase distribution of HEK293 cells (bottom panel). The data shown are the mean of three independent experiments. The data were analyzed as indicated in Materials and Methods Section. The standard deviation (T bar) and the statistical significance (*p* value) are reported.

To evaluate the phenotypical effects of the BWS mutant R316W-p57 (Figure 1A), site-directed mutagenesis was performed to introduce this genetic change into the coding sequence of wild-type (WT) human p57 cloned in pcDNA3.1 plasmid. Subsequently, the empty plasmid (CTRL) and those encoding WT-p57 and the variant protein, respectively, were transfected in triplicate in the HEK293 cell line to evaluate the effect of the exogenously expressed proteins on the proliferation rate. The cells were collected, counted, and the cell extracts were analyzed by means of immunoblotting after 24 h and 48 h to verify equal levels of transfection (Figure 1B). The analysis of the proliferation rate showed a clear inhibitory effect of the WT protein on the cell proliferation rate (Figure 1C, upper histogram). In parallel, the cell cycle phase distribution of HEK293 that had been transfected for 48 h with WT-p57 showed a G0/G1 phase arrest (Figure 1C, lower histogram). Figure 1C also reports the results of the analysis of the effects of R316W-p57 on cell proliferation (upper histogram) and on cell cycle phase distribution (lower histogram). The BWS mutant showed a slightly higher, although not statistically significant, proliferation rate compared to the WT protein. The FACS analysis reported that R316W-p57 has a minor effect on cell cycle distribution compared to WT-p57, being more comparable to cells treated with the empty vector.

### 2.2. Subcellular Localization of R316W-p57

**Figure 2:**
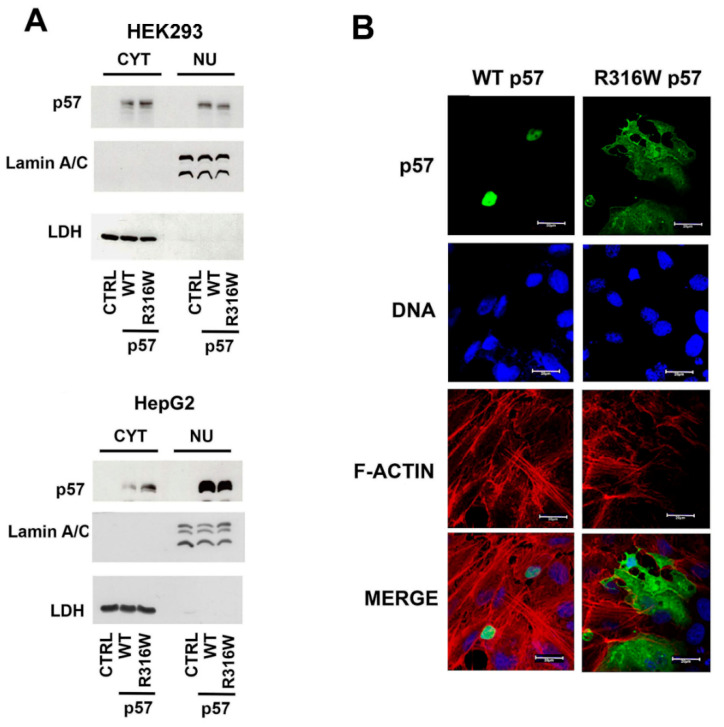
Subcellular localization of human WT- and R316W-p57. (**A**) WB analysis of the cytosolic and nuclear content of human p57 in HEK293 and HepG2 cell lines after 24 h of transfection with the empty vector (CTRL) and the plasmids encoding WT- and R316W-p57. Lamin A/C and LDH were analyzed to confirm the proper separation of nuclear and cytosolic fractions and as control for equal protein loading. (**B**) The subcellular localization of the WT and the mutated protein was investigated using immunofluorescence. HepG2 cells were stained with Alexa Fluor 488 (anti-p57 Ab, green), Hoechst 33342 (DNA, blue), and phalloidin (F-ACTIN, red). Representative images of cells overexpressing WT- and R316W-p57 were acquired using confocal microscopy with a 63x objective and are shown in panel (**B**). Scale bar 20 µm. For further details refer to the Materials and Methods Section.

Being that the localization of CIP/Kip proteins is essential for their activity, we investigated the subcellular localization of the R316W mutated p57 compared to the WT protein. Thus, the nuclear and the cytoplasmatic levels of the exogenously expressed proteins were evaluated by immunoblotting of protein extracts of the HEK293 and HepG2 cells transfected for 24 h with the plasmids encoding WT-p57, R316W-p57, and the empty vector. In both cell lines the WT protein localized mostly in the nucleus, and, particular in HepG2 cells, p57 was barely detectable in the cytoplasm. The difference (nuclear p57 versus cytosolic p57) between the transfected cell lines indicates a cellular context-specific behavior. Interestingly, the localization of the BWS-related R316W-p57 mutant presented an inverted ratio of the cytoplasmic/nuclear content with respect to the WT protein in the HEK293 cells (Figure 2A). A clear R316W-p57 increase in the HepG2 cytoplasm was also observed (Figure 2A). As shown in Figure 2B, confocal microscopy analyses performed on the HEPG2 cells clearly confirmed R316W-p57 mutant relocalization in the cytoplasm compared to WT-57.

### 2.3. The Nuclear Localization of p57 Depends on the C-Terminal Domain

**Figure 3:**
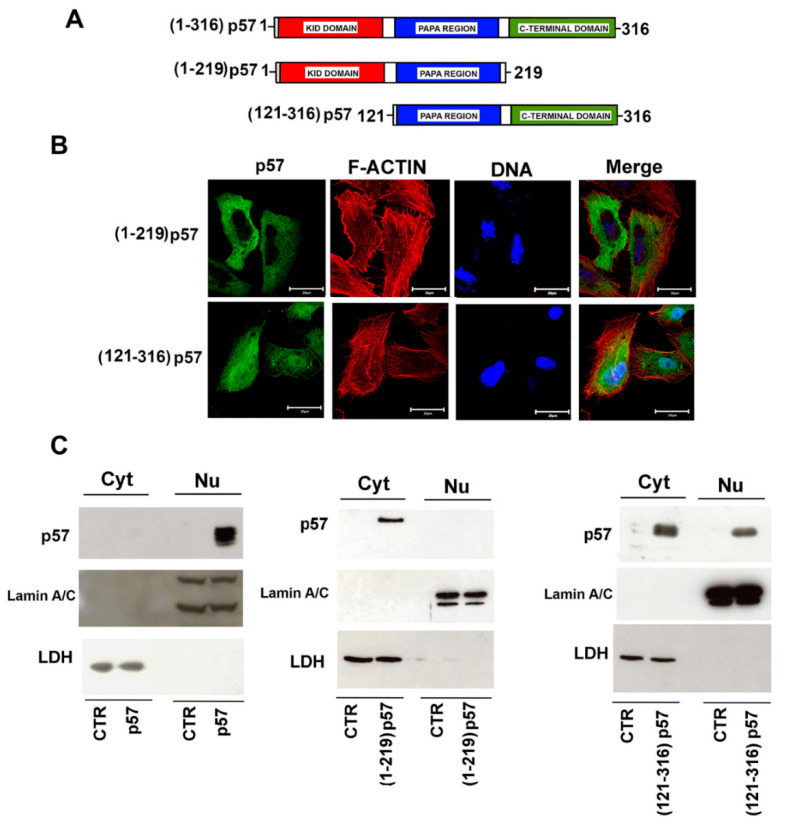
Nuclear localization of p57 is dependent on the C-terminal domain. (**A**) Schematic representation of the FL WT-p57 and the two N-terminal [(1–219)p57] and C-terminal [(121–316)p57] constructs. (**B**) The cellular localization of the exogenously expressed protein fragments was investigated using immunofluorescence as described in Figure 2B. Representative immunofluorescence images of cells transfected with the plasmids encoding the N-terminal and C-terminal p57 fragments were reported. p57 (green), DNA (blue), and F-ACTIN (red). Scale bar 20 µm. For further details, refer to the Materials and Methods Section. (**C**) WB analysis of the cytosolic and nuclear content of p57 in HepG2 cells after 24h of transfection with the plasmids encoding FL WT-p57 (p57) and the two fragments (1–219)p57 and (121–316)p57. Lamin A/C and LDH were analyzed to confirm the proper separation of nuclear and cytosolic fractions and as control for equal protein loading.

As described in the Introduction, for simplicity, the p57 sequence might be divided in two domains, i.e., the N-terminal domain (that includes the KID region) and the C-terminus. To identify the protein region involved in p57 nuclear localization, we subcloned the sequence of the p57 cDNA to generate two independent p57 fragments, each inserted in a pcDNA3.1 expression plasmid. The first construct includes 690 bps and encodes the N-terminal region, i.e., from amino acids 1 to 219. The second embraces 624 bps and encodes the C-terminal region from amino acids 121 to 316. Both the constructs include the PAPA region, the central domain of the protein; thus, the differences eventually observed will be dependent only on the KID or on the C-terminal p57 domain (Figure 3A). The subcellular localization of the two protein constructs was evaluated using immunofluorescence and immunoblotting. As showed in Figure 3B,C, the N-terminal fragment (1–219)-p57 mainly localizes in the cytoplasm, differently from the full-length (FL) protein (WT-p57) (Figure 1B and Figure 3C). Conversely, the C-terminus fragment (121–316)-p57 is distributed both in the nucleus and in the cytoplasm (Figure 3B,C), demonstrating that nuclear localization depends on this protein domain. In summary, the data obtained suggests that one or more p57 NLS(s) reside between residue 220 and residue 316.

### 2.4. A Putative NLS Sequence Occurs at the End of the p57 C-Terminal Domain

**Figure 4:**
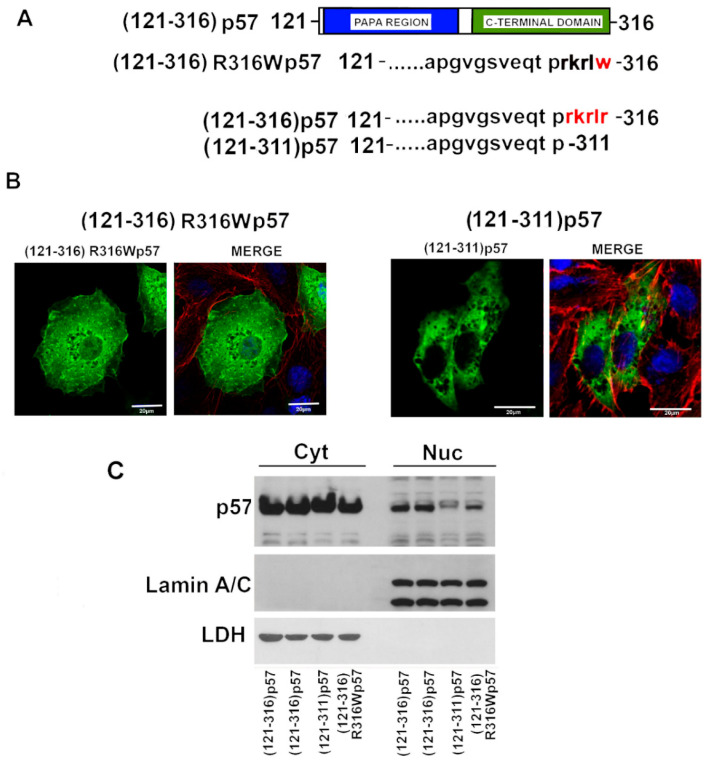
Cellular distribution of the (121-316)p57, (121-316)R316Wp57, and the truncated fragment (121–311)p57. (**A**) Schematic representation of the human C-term (121–316)p57 fragment, the mutagenized (121–316)R316Wp57, and the truncated (121–311)p57 protein fragments. (**B**) Representative immunofluorescence images of HepG2 cells transfected with the plasmids encoding the indicated proteins listed in panel A. p57 (green), DNA (blue), and F-ACTIN (red). Scale bar 20 µm. For further details refer to the Materials and Methods Section. (**C**) WB analysis of the cytosolic and nuclear content of p57 in HEK293 after 24h of transfection with the plasmids represented in panel A. Lamin A/C and LDH were analyzed to confirm the proper separation of nuclear and cytosolic fractions and as control for equal protein loading.

To further investigate the molecular basis of the altered localization of the BWS-associated R316W-p57 mutant, we decided to perform an in silico analysis of the C-terminus primary sequence, which is where the residue change occurs. The study revealed that arginine 316 is localized at the end of a strongly basic arginine–lysine rich sequence (312-RKRLR-316) that has a high homology with a canonical NLS motif (Figure 4A) [24]. Since this sequence could be crucial for nuclear import, we constructed two C-terminus p57 mutants, one bearing the R316W change and a second in which the putative NLS was removed by introducing a stop codon in place of R312 [(121–311)-p57]. The cellular distribution of R316W C-term [(121–316)-R316Wp57] and (121–311)-p57 fragments was investigated using transfection and subsequent confocal microscopy (Figure 4B) and Western blotting (Figure 4C) analyses. Both the (121–316)-R316Wp57 and (121–311)-p57 C-term fragments showed a reduction of the p57 nuclear content compared to the (121–316)-p57 protein fragment. In particular, (121–311)-p57 presented a clearly predominant cytosolic localization, as shown by the confocal microscopy analysis. These data strongly suggest the possible occurrence of a p57 putative NLS that includes the last five protein amino acids (Figure 4B,C). In conclusion, we demonstrated that the basic amino acid region from Arg312 to Arg316 is required for the nuclear localization of the C-terminal fragment of p57.

### 2.5. Identification of p57 NLS

**Figure 5:**
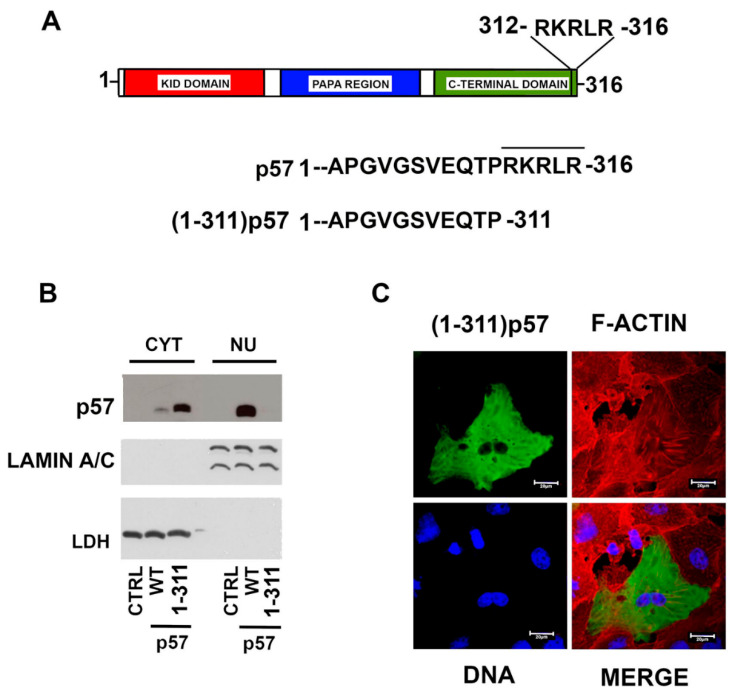
Relevance of the last five p57 residues in the nuclear localization of the protein. (**A**) Schematic representation of FL WT-p57 and (1–311)-p57. (**B**) WB analysis of p57 cytoplasmic/nuclear distribution in HEPG2 cells transfected for 24 h with the empty vector (CTRL) and the plasmids encoding the WT- and (1–311)-p57. Lamin A/C and LDH were employed to confirm the proper separation of nuclear and cytosolic fractions and as control for equal protein loading. (**C**) Representative immunofluorescence image of the overexpressed (1–311)p57 in HepG2 cells. p57 (green), DNA (blue), and F-ACTIN (red). Scale bar 20 µm. For further details refer to the Materials and Methods Section.

Since we demonstrated that the nuclear localization of the C-terminal fragment of p57 relies on the last five amino acids, we expected that the deletion of this sequence in the full-length protein might determine the complete exclusion of the protein from the nucleus. Accordingly, we performed a mutagenesis experiment introducing a stop codon at Arg312 in the sequence codifying the FL WT protein, as we did for the C-terminal fragment. We defined this protein as (1–311)-p57 (Figure 5A). Subsequently, HepG2 cells were transfected for 24h with the empty vector and the plasmids encoding FL WT-p57 and (1–311)-p57. The localization of the proteins was then analyzed using immunoblotting and confocal microscopy. The immunoblotting analyses clearly highlighted the complete cytoplasmic localization of (1–311)-p57, showing an inverted nuclear/cytoplasmic ratio compared to the mostly nuclear FL WT-p57 (Figure 5B). It is of note that in the immunoblotting of Figure 4C, a small amount of (121–311)-p57 is localized in the nucleus, while (1–311)-p57 is completely cytoplasmic (Figure 5B). This suggests that the initial 120 aa of p57 might affect protein localization/interaction in the cytosol. The immunofluorescence analysis of the (1–311)-p57 truncated mutant directly confirmed the biochemical results (Figure 5C). In brief, the sequence corresponding to RKRLR from the amino acid R312 to R316 appeared to be absolutely necessary for the nuclear localization of p57, allowing us to definitely identify the occurrence of an NLS motif localized in the C-terminus of the CDK inhibitor (Figure 5A).

## 3. Discussion

Mutations of the *CDKN1C* gene have been found in a number of congenital growth syndromes including BWS, IMAGe, and RSS, strongly supporting the importance of p57 for correct development [19,22,25]. In particular, BWS can be caused by the epigenetic and genetic deregulation of a cluster of genes in the locus 11p15.5 where *CDKN1C* maps [26]. Phenotypically, individuals with BWS display overgrowth and several congenital malformations, including macroglossia, abdominal wall defects, and tumour predisposition [27]. RSS and IMAGe are clinically opposite to BWS [20]. RSS patients show severe postnatal growth retardation, skeletal abnormalities and other clinical signs partially overlapping with those observed in subjects affected by IMAGe syndrome [28]. Notably, the mutational status of *CDKN1C* assumes remarkable relevance, especially for IMAGe syndrome where the clinical diagnosis is established through the identification of a missense variant in the maternal *CDKN1C* gene [29]. Similarly, in RSS, in the absence of a clear clinical characterization, genetic molecular analysis, including searching for *CDKN1C* mutations, can allow for a more accurate management of the pathology [28]. Of interest, in BWS individuals that do not have epigenetic alterations, *CDKN1C* gene mutations are reported, reaching more than 50% of familial BWS cases [21]. *CDKN1C* alterations can be classified into two major families. *CDKN1C* variations identified in subjects with BWS should be mechanistically considered “loss of function” mutations [4,27,30], while the genetic changes described in subjects with IMAGe and RSS syndromes might be viewed as “gain of function” mutations [19,25,31]. Importantly, IMAGe and RSS p57 changes cluster in a small region of the C-terminal PCNA-binding domain [19,25]. Due to the phenotypes of both syndromes, these mutations have been associated with increased protein stability secondary to the loss of binding with PCNA [19,32,33] resulting, in turn, into hypoproliferative features. The majority of mutations reported for *CDKN1C* have been identified in BWS subjects and, differently from IMAGe and RSS, these genetic changes were found throughout the gene sequence. In general, missense mutations affect the functionality of the KID domain, which is necessary for the inhibition of cell proliferation [34,35], while many of the frameshift and nonsense BWS *CDKN1C* mutations do not modify the activity of the KID [22]. The most accepted mechanistic hypothesis for explaining their pathogenic effect is that truncated mutants, after losing the C-terminal domain, cannot translocate into the nucleus and, in turn, cannot inhibit the Cyclin-CDKs complexes, becoming functionally inactive [22]. This hypothesis, however, needs to be substantiated by a direct identification of sequence(s) that are absolutely necessary for human p57 nuclear localization. So far, the characterization of the human p57 C-terminus domain has been partially based on homology with human p21^Cip1/WAF1^, p27^Kip1^, and mouse p57 sequences [1,2]. In particular, while the region required for nuclear entry in mouse p57 was experimentally mapped at residues 281–348 [1], the recognition of an NLS was obtained by homology with human p27Kip1 and was putatively localized in mouse p57 at residues 309–312 (KRKR). In human p57, in turn, this motif should correspond to aa 278–281 (KRKR). However, no direct investigations have been conducted to identify the sequence of human p57 required for nuclear localization, despite its mechanistic key relevance in BWS-associated mutations.

In this context, when we examined BWS missense mutations, we were surprised that one variant p.Arg316Trp (R316W-p57) showed a change on the last residue of the protein [23]. Since the mutation occurs in a sequence of lysine–arginine repeats typical of an NLS [24], we hypothesized that it might be an interesting portion of a still unrecognized human p57 NLS sequence. To evaluate this view, the cellular distribution of the mutant was investigated and a partial relocalization in the cytosol was evidenced. The analysis of two transfected p57-fragments (1–219 and 121–316) then demonstrated that the NLS was localized between residues 220 and 316. These results substantially strengthen the data reported for the mouse protein, although the C-terminus of the mouse protein was only partially homologous to its human counterpart. In addition, when we substituted the Arg316 with Trp on the C-terminal fragment, we observed its partial cytosolic accumulation. This finding confirmed the importance of the mutation in relocating the fragment from the nucleus to the cytosol. Therefore, the obtained results were in line with the hypothesis that the nuclear transport of the protein was mediated by a putative NLS localized in the C-terminal region of the protein and that the correct end of the protein might be important for nuclear entry. The data however do not definitely distinguish between a key role of arginine as part of a putative NLS or a negative role of tryptophan in hampering nuclear localization. By analyzing the primary structure of the protein, a basic sequence RKRLR from Arg312 to Arg316 that have the molecular features of a canonical NLS is recognizable. Thus, we hypothesized that these five amino acids were responsible for the nuclear import of the protein. To prove that, we generated a mutant lacking the amino acids from R312 to R316 on both the C-terminal fragment [(121–311)-p57] and on the full-length protein ((1–311)-p57), and we analyzed the respective subcellular distribution after overexpression. The immunoblotting and the immunofluorescence results confirmed the exclusion from the nucleus for both mutants.

## 4. Materials and Methods

### 4.1. Cell Culture, Cell Extracts Preparation and Immunoblotting Analyses

Human HEK293 and HepG2 cell lines (ATCC, Manassas, VA, USA) were cultured in DMEM HG (Gibco, Thermo Fisher Scientific, Waltham, MA, USA), supplemented with 10% fetal bovine serum (FBS, Invitrogen, Thermo Fisher Scientific), 100 U/mL benzylpenicillin, 100 mg/L streptomycin (Gibco), and cultured in a humidified incubator with 5% CO_2_ at 37 °C. Total and fractionated cell protein extracts were obtained and analyzed as previously reported [36,37]. The efficiency of the nuclear and cytosolic separation was tested through immunoblotting of LAMIN A/C and LDH, respectively. The following primary antibodies were employed: anti-p57 (mouse monoclonal), anti-p57 (rabbit polyclonal), anti-LAMIN A/C (mouse monoclonal), anti-LDH (mouse monoclonal), and anti-β-actin (mouse monoclonal), all purchased from Santa Cruz Biotechnology, Inc., Heidelberg, Germany.

### 4.2. Plasmid Preparation and Transfection

Plasmids were prepared using the DNA coding sequences of the human FL p57, N-term, and C-term p57 subcloned in pcDNA3.1. The QuikChange II Site-Directed Mutagenesis Kit (Agilent Technologies, Inc., Santa Clara, CA, USA) was used for mutagenesis reactions following the manufacturer’s instructions. Specifically, pcDNA3.1 containing the following mutations were prepared: R316W-p57, (1–311)-p57, (121–316)-p57, (121–316)-R316W-p57, (121–311)-p57, and (1–219)-p57. Each mutagenesis product was confirmed by direct sequencing [38]. HepG2 and HEK293 cells, at 60–70% confluence, were transfected with the plasmid using Lipofectamine 3000 (Invitrogen) according to the manufacturer’s instructions [39,40]. Control cells were transfected with the empty vector.

### 4.3. Analysis of Proliferation Rate and Cell Cycle Distribution

For the analysis of the proliferation rate, HEK293 cells were cultured in 6 well plates with 1.5 × 10^5^ cells per well. After 24 h, cells were transfected in triplicate with empty pcDNA3.1 vector (CTRL) and with the constructs containing the coding sequence for p57 and R316W-p57, using 500 ng of the respective plasmid for each transfection. Cells were trypsinized, collected, and counted using a Burker chamber at time T0 (untransfected), T1 (24 h), and T2 (48 h). The mean values ± standard deviation (T-bar) were plotted in a histogram diagram. Lysates obtained from the cell pellets were analyzed by Western blotting using anti-p57 and anti-actin antibodies to verify a comparable level of transfection and equal loading. Cell cycle distribution analysis was performed as previously reported in Stampone et al., 2020 [40], and the results were plotted in a histogram diagram as mean values ± standard deviation.

### 4.4. Immunofluorescence Microscopy

Immunofluorescence microscopy was performed as in Bencivenga et al., 2021 [41]. Cells were grown in 8-well tissue culture chambers (Ibidi, Gräfelfing-Bayern, Germany) and transfected for 24 h with the plasmids indicated before. Cells were washed with PBS and fixed with 4% (*w/v*) paraformaldehyde for 15 min at room temperature. Subsequently, cells were washed 3 times with PBS and permeabilized for 10 min with PBS-0.1% Triton X-100, before incubating them with the blocking buffer for 1 h. Cells were the incubated with anti-p57 Ab overnight at 4 °C. Alexa Fluor 488-conjugated secondary antibody (Abcam, Cambridge, UK) was added for 1h. Cells were then stained with Phalloidin CruzFluo 555 Conjugate (Santa Cruz Biotechnology) for 20 min and Hoechst 33342 Trihydrochloride Trihydrate (Thermo Fisher Scientific) for 10 min. Fluorescent images were obtained using a Carl Zeiss LSM 700 confocal laser scanning microscope (Zeiss LSM 700, Zeiss, Oberkochen, Germany) through a 63×/1.4 PlanApo oil immersion objective.

### 4.5. Statistical Analysis

Experimental data were expressed as mean ± SD. The statistical significance was calculated using a Sample T test after verifying the normality (Shapiro–Wilk and Kolmogorov–Smirnov tests) and the homoscedasticity (Spearman’s test) of the data using the software GraphPad Prism 9.1.2. A value of *p* < 0.05 was considered statistically significant.

## 5. Conclusions

Although our study does not allow us to rule out the occurrence of additional NLSs, including the already proposed in the region 278–281, in particular, it argues for the pivotal role of the C-terminal RKRLR sequence in the nuclear localization of p57. This unprecedented observation has various consequences. First, being p57 mostly a nuclear protein, the identification of a necessary sequence for its nuclear entry is important in understanding the precise activity, interaction, trafficking and turn-over of p57. Second, a large number of *CDKN1C* mutations causes frameshifts or the synthesis of a truncated proteins. Thus, since the identified NLS is localized at the C-terminus, these mutants should mostly relocalize (or at least in large part) to the cytosol. It is important to stress that some BWS-associated *CDKN1C* mutations, produce protein variants that still maintain several p57 functional domains, including the KID and the previously described NLS. One of these is the p.Ser282X variant that has been found in several BWS individuals [21,23]. As matter of fact, the loss of function of this p57 mutant could be associated to the generation of a truncated protein missing the last 34 amino acids and thus possibly localized in the cytosol. The occurrence of the BWS-related mutation p.Pro302Leufs*19 [21] further narrowed the analysis window on the last 15 amino acids of the protein, suggesting that the absence of the identified nuclear localization signal might be important in explaining the pathogenicity of numerous *CDKN1C* genetic alterations. In actual fact, the cytosolic interactions of p57 and its degradation mechanism have not been precisely determined. In the same context, since, as we have reported, the protein is strongly phosphorylated, an increased cytosolic relocalization might affect its post-translational modifications and, in turn, its functions. Further experiments are under development for evaluating the relevance of our discovery in the mechanistic development of human syndromes associated with *CDKN1C* alterations.
